# Secreted frizzled-related protein 4 expression is positively associated with responsiveness to Cisplatin of ovarian cancer cell lines *in vitro* and with lower tumour grade in mucinous ovarian cancers

**DOI:** 10.1186/1471-2121-13-25

**Published:** 2012-10-08

**Authors:** Uttara Saran, Frank Arfuso, Nikolajs Zeps, Arunasalam Dharmarajan

**Affiliations:** 1 School of Anatomy and Human Biology, Faculty of Life and Physical Sciences, The University of Western Australia, 35 Stirling Highway, Perth, Crawley 6009, Western Australia; 2 St John of God HealthCare, Subiaco, 6008, Western Australia; 3 School of Surgery, The University of Western Australia, 35 Stirling Highway, Crawley, 6009, Western Australia; 4 School of Pathology and Laboratory Medicine, The University of Western Australia, 35 Stirling Highway, Crawley, 6009, Western Australia; 5 School of Biomedical Sciences, Faculty of Health Sciences, Curtin University and Curtin Health Innovation Research Institute (CHIRI), GPO Box U1987, Perth, 6845, Western Australia

**Keywords:** Secreted frizzled-related protein 4, Ovarian cancer, Cisplatin, Chemoresistance, Chemosensitivity, Tumour biopsy

## Abstract

**Background:**

Ovarian cancer is one of the most lethal malignancies in women, as it is frequently detected at an advanced stage, and cancers often become refractory to chemotherapy. Evidence suggests that dysregulation of pro-apoptotic genes plays a key role in the onset of chemoresistance. The secreted Frizzled-Related Protein (sFRP) family is pro-apoptotic and also a negative modulator of the Wnt signalling cascade. Studies have demonstrated that the re-expression of sFRPs, in particular sFRP4, is associated with a better prognosis, and that experimentally induced expression results in cell death.

**Results:**

*In vitro* experimental models determined that sFRP4 was differentially expressed in chemosensitive (A2780) and chemoresistant (A2780 ADR and A2780 Cis) ovarian cell lines, with chemosensitive cells expressing significantly higher levels of sFRP4. Transfection of the chemoresistant cell lines with sFRP4 significantly increased their sensitivity to chemotherapy. Conversely, silencing of sFRP4 expression in the chemosensitive cell line resulted in a corresponding increase in chemoresistance. Comparison of sFRP4 expression in tumour biopsies revealed a positive trend between sFRP4 expression and tumour grade, with mucinous cyst adenocarcinomas exhibiting significantly decreased sFRP4 levels compared to mucinous borderline tumours.

**Conclusions:**

This study indicates a role for sFRP4 as a predictive marker of chemosensitivity in ovarian cancer and suggests that this pathway may be worth exploiting for novel therapies.

## Background

Ovarian cancer is the fifth leading cause of cancer deaths in women, with epithelial ovarian carcinomas being the most prevalent type diagnosed [1-4]. Chemoresistance, a common development in ovarian carcinomas, is a major hurdle that significantly hinders treatment success [5,6]. Recent evidence suggests that the dysregulation of pro-apoptotic genes is a key factor for the onset and maintenance of chemoresistance [5,7]. One of such pro-apoptotic gene families is the Secreted Frizzled-Related protein (sFRPs) family, an antagonist of the Wnt signalling pathway. sFRPs have a shared homology with Frizzled receptors and are thought to be able to impede Wnt-Fz interactions, thereby blocking Wnt activity in cells. Wnts have been established to play essential roles during foetal development [1,8-14] as well as maintaining homeostasis in adult tissues [15]. However, aberrant canonical Wnt signalling has been widely described in cancer and has been implicated as an important contributor to tumour development [1,8,12]. Several studies have reported a correlation between up-regulation of sFRP expression, in particular that of sFRP4, and apoptosis in various tissues [16-22]. The over-expression of sFRPs has been shown to decrease β-catenin levels within the cells, thus increasing their susceptibility to pro-apoptotic stimuli [23-25]. While restoration or up-regulation of sFRP expression in cancer cells was shown to attenuate their tumourigenic behaviour by inhibiting Wnt signalling and inducing apoptosis [9,25-31], hypermethylated silencing of sFRP genes tended to increase with tumour progression and invasiveness [29,32,33].

In this study we investigated the effects of over-expressing or silencing of sFRP4 in ovarian cancer lines with different chemosensitivity in an *in vitro* model. We further examined sFRP4 expression in human ovarian tumours to assess if its expression could be correlated with clinico-pathological features consistent with a proposed role for loss being a contributor to chemoresistance.

## Results

### Differential expression of sFRP isoforms in ovarian cancer cell lines

**Figure 1:**
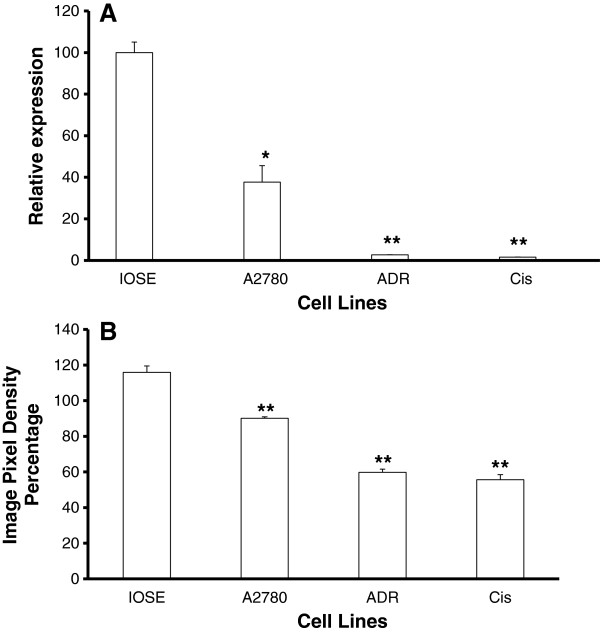
**Comparison of quantitated sFRP4 expression across the four ovarian cell lines. **The expression of sFRP4 in cancerous cell lines was compared to IOSE (normal) ovarian cell line. (**A**) sFRP4 mRNA expression across four cell lines. (**B**) sFRP4 protein expression across four cell lines. Values represent means for each group ± SEM (*p < 0.05; ** p < 0.001; one way ANOVA and LSD).

The expression profiles of all 5 isoforms of sFRP were determined in the four cell lines used in this study. sFRP2 was not detected in any of the cell lines (data not shown), and only sFRP4 was differentially expressed between the chemosensitive (A2780) and chemoresistant cancer cell lines (A2780-ADR and A2780-Cis); with A2780 expressing significantly higher mRNA levels of sFRP4 in comparison to the A2780-ADR and A2780-Cis (Figure 1A). Western blot analysis of sFRP4 protein levels determined that the normal cell line IOSE expressed significantly higher levels of sFRP4 (p < 0.001) compared to the cancer cells. Furthermore, the chemosensitive A2780 cells also exhibited significantly higher levels of sFRP4 (p < 0.001) compared to the chemoresistant cell lines (Figure 1B). A representative image of the Western blot is shown in Additional file 1: Figure S1.

### sFRP4 expressing cells are selectively killed by Cisplatin

**Figure 2:**
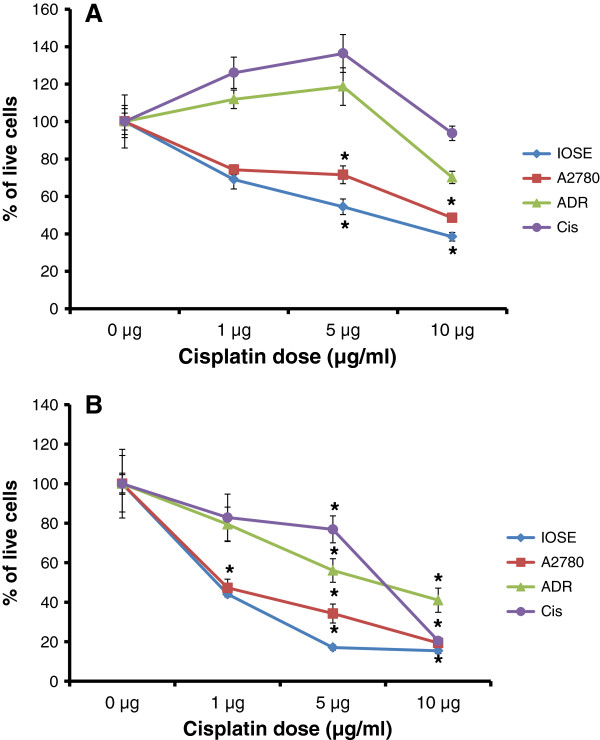
**Graphical representation of cell viability following various doses of Cisplatin treatment with time. **(**A**) Percentage of live cells after 24 hours treatment with 3 doses of Cisplatin (1, 5 and 10 μg). (**B**) Percentage of viable cells following 48 hours of Cisplatin treatment. Values represent means for each group ± SEM (* p < 0.001 one way ANOVA and LSD).

Analysis of MTS cell viability assays following Cisplatin treatment demonstrated that both the IOSE and A2780 cells, which were shown to express more sFRP4, had a significant reduction (p < 0.001) in cell viability compared to untreated controls within 24 h for all three treatment doses administered, and continued to exhibit decreased viability for the remaining time points (Figure 2A). The chemoresistant cell lines continued to proliferate, but a significant reduction in cell viability was shown at 48 h after treatment (Figure 2B).

**Figure 3:**
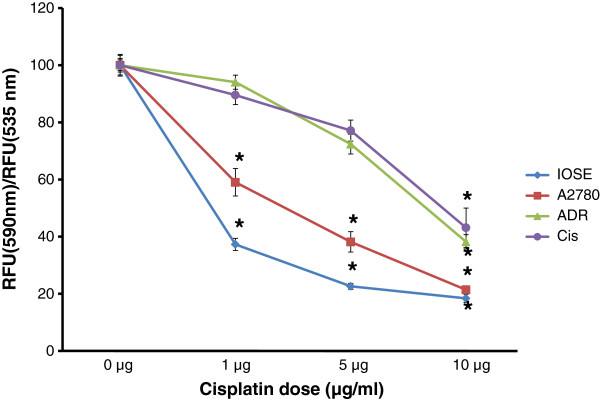
Quantification of cell death by JC-1 analysis (red/green fluorescence ratio) with 3 doses of Cisplatin (1, 5 and 10 μg) for 48 hours. Values represent means for each group ± SEM (* p < 0.001 one way ANOVA and LSD).

Following Cisplatin treatment, only IOSE cells demonstrated significant mitochondrial membrane depolarization in all treatment groups within 24 h after treatment. Although the chemosensitive cell line A2780 demonstrated decreased cell viability in all three treatment groups, cell death was detected only at the treatment dose of 10 μg/ml. In contrast, cell death was detected in the chemoresistant cell lines only after 48 h treatment (p < 0.001) (Figure 3), suggesting that their lower sFRP4 levels could potentially be one of the factors influencing the delayed response of these cells.

**Figure 4:**
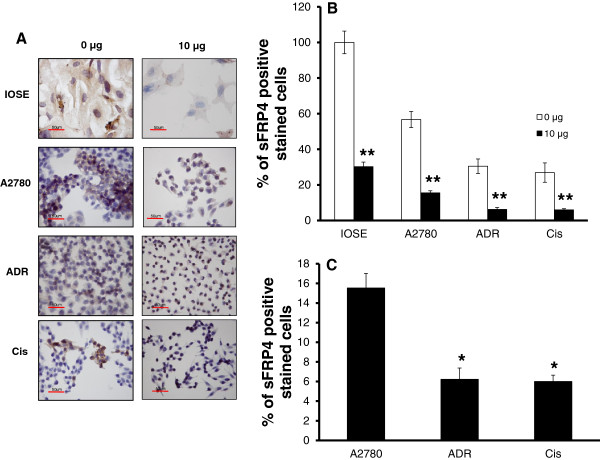
**(A) Light microscope images (400X) of each cell line before and after Cisplatin treatment.** sFRP4 protein stained brown with DAB and counterstained with haematoxylin for contrast (Scale bar = 50 μm). (**B**) Comparison of sFRP4 protein expression between untreated and treated cells of all four cell lines. (**C**) Quantification of the proportion of cells stained positive for sFRP4 following Cisplatin treatment in chemosensitive cell line A2780 vs. Chemoresistant cell lines ADR and Cis. Values represent the mean for each group ± SEM. (* p < 0.01; ** p < 0.001 one way ANOVA and LSD).

IHC revealed that sFRP4 expression could not be detected in the majority of surviving cells. Compared to untreated controls, the percentage of IOSE cells still expressing sFRP4 had decreased by 70% following treatment with Cisplatin (10 μg/ml) for 48 h (Figure 4A, B). Similarly, the chemosensitive cell line A2780 also demonstrated a reduction of about 45% of its sFRP4 expressing cell population following treatment. In comparison, the chemoresistant cell lines demonstrated a greater percentage of live cells following treatment and only showed a reduction of 25% in the number of cells expressing sFRP4. Additionally, the difference in numbers of sFRP4 expressing cells between chemosensitive and chemoresistant cell lines was highly significant (p < 0.01) (Figure 4C).

### Over expression of sFRP4 enhanced chemosensitivity of tumour cells

**Figure 5:**
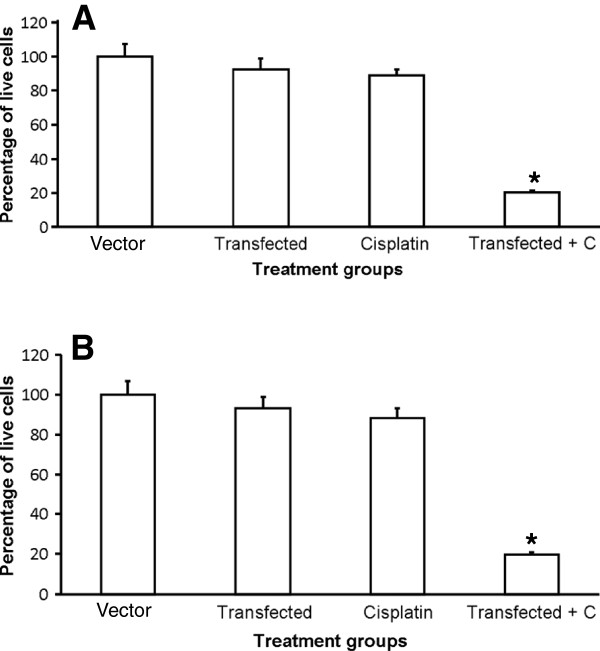
**Effect of transfection with sFRP4 plasmid and subsequent Cisplatin treatment for 24 hours. **(**A**) A2780-ADR live cell number following transfection with sFRP4 plasmid and subsequent Cisplatin treatment for 24 hours. (**B**) A2780-Cis live cell number following transfection with sFRP4 plasmid and subsequent Cisplatin treatment for 24 hours. Values represent means for each group ± SEM (* p < 0.001; one way ANOVA and LSD).

Since chemotherapy appeared to be selectively killing sFRP4 expressing cells, we sought to test this relationship further by transfecting the chemoresistant cell lines with a plasmid expressing sFRP4 before treating them with Cisplatin. Results showed that increasing sFRP4 expression of the chemoresistant cell lines A2780-ADR and A2780-Cis (Additional file 2: Figure S2A) before treatment with Cisplatin for 24 h resulted in a highly significant decrease in cell viability (p < 0.001) compared to control groups (Figure 5A and B respectively).

### Down regulation of sFRP4 decreased chemosensitivity of tumour cells

**Figure 6:**
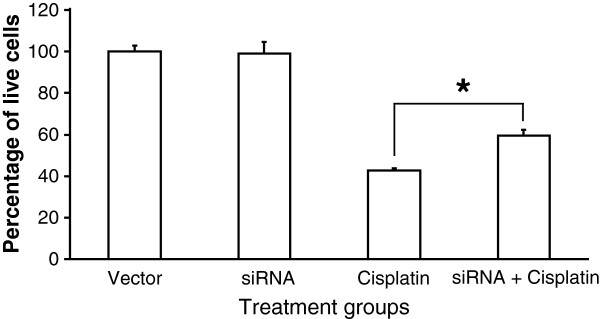
**Effect of silencing of sFRP4 and subsequent Cisplatin treatment. **Graphical representation of A2780 live cell number quantified following silencing of sFRP4 and subsequent Cisplatin treatment for 24 hours. (* p < 0.001; one way ANOVA and LSD).

Data generated from cell viability and cell death assays showed an inverse relationship between sFRP4 expression and the response of tumour cells to Cisplatin treatment. To further investigate this correlation, the sFRP4 expression levels of the chemosensitive cell line A2780 was knocked down using siRNA. The A2780 cells treated with sFRP4-siRNA demonstrated a 40% decrease in sFRP4 mRNA and protein expression compared to control cells (Additional file 2: Figure S2B and C). Results showed that the control group subjected to Cisplatin treatment exhibited a 60% reduction in cell viability compared to untreated controls. However, the cells subjected to sFRP4-siRNA treatment only exhibited a 40% reduction in cell viability following treatment with Cisplatin compared to control cells (Figure 6). The relative increase in cell viability of Cisplatin treated sFRP4-siRNA cells indicates that decreasing sFRP4 in chemosensitive cells caused more resistance to Cisplatin.

### Differential sFRP4 expression was correlated to its interaction with the Wnt/β-catenin signalling pathway

**Figure 7:**
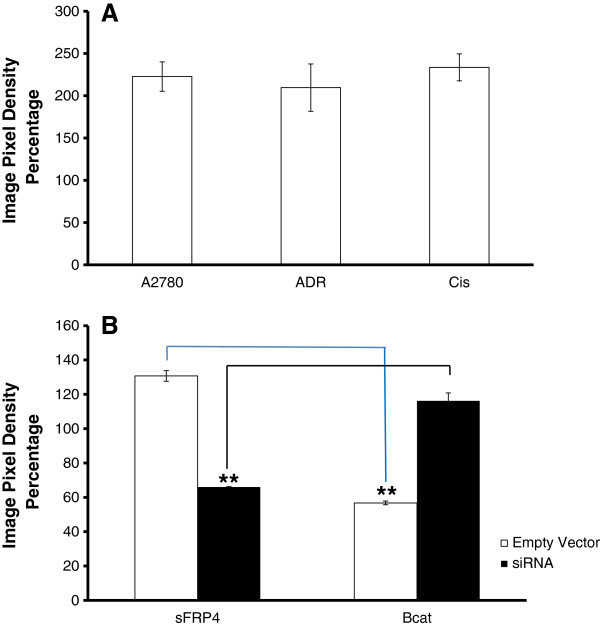
**(A) β-catenin protein expression across the three cancerous cell lines. **(**B**) Graphical representation demonstrating inverse sFRP4 and β-catenin protein expression following siRNA treatment in the chemosensitive cell line A2780. Values represent the mean for each group ± SEM. (** = p < 0.001 one way ANOVA and LSD).

Western blot analysis for β-catenin protein showed that in contrast to sFRP4, all three cell lines exhibited elevated levels of β-catenin, indicating the presence of an active Wnt pathway (Figure 7A; a representative image of the Western blot is shown in Additional file 3: Figure 3A). Although the un-transfected chemoresistant cell lines showed relatively high β-catenin expression, this expression could not be detected in the sFRP4-transfected cells. Additionally, the 40% decrease of sFRP4 expression in chemosensitive A2780 cells due to silencing was accompanied by a corresponding increase in β-catenin expression (Figure 7B; representative images of the Western blots are shown in Additional file 3: Figure S3B), confirming a functional relationship between sFRP4 and the Wnt/β-catenin signalling pathway in this cell line.

### Association of sFRP4 and stage in primary mucinous ovarian tumours

**Figure 8:**
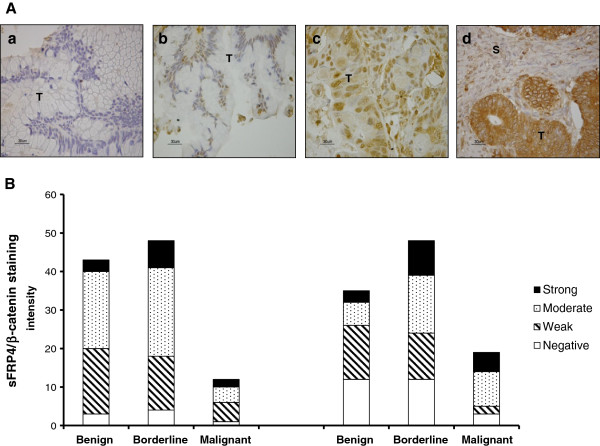
**(A) Examples of TMA scoring based on staining intensity. **Light microscope images of mucinous ovarian tumour tissue (T) and stroma (S) from the TMAs. Tissue samples expressing sFRP4 are stained brown with DAB and counterstained with haematoxylin. Images show examples of (**a**) Negative (**b**) Weak (**c**) Moderate and (**d**) Strong staining. Scale bar = 30 μm. (**B**) Graphical representation of the staining intensity quantified for sFRP4 expression and β-catenin expression respectively, following histological quantification of the tumour cores into benign, weak, or strong. The y axis enumerates the staining intensity within each group of tumours.

**Figure 9:**
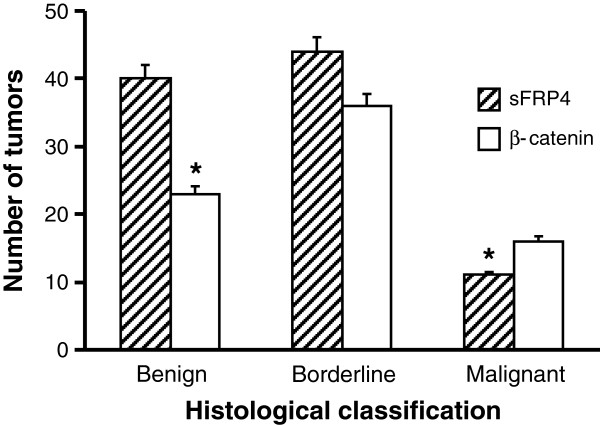
**Graphical representation of the quantified proportion of tumours expressing sFRP4 and β-catenin based on histological classification of mucinous tumours as benign, borderline and adenocarcinomas. **Values represent the mean for each group ± SEM. (* p < 0.001).

We performed sFRP4 (Figure 8A) and β-catenin IHC on primary mucinous ovarian cancer biopsies in tissue microarrays (TMAs) comprising 104 primary mucinous ovarian tumours. The TMA sections were subjectively classified into one of four categories depending on the percentage and intensity of the cellular sFRP4 staining: negative, weak, moderate or strong (Figure 8A). The primary mucinous ovarian tumours were classified into three sub-types; namely benign, borderline, and adenocarcinomas, and the proportion of sFRP4 and β-catenin was quantified for each (Figure 8B). Analysis revealed that the majority of benign and borderline tumours exhibited sFRP4 expression, while adenocarcinomas demonstrated a significantly reduced sFRP4 expression compared to borderline and benign tumours (p < 0.001) (Figure 9). An inverse trend was observed for β-catenin, with benign tumours expressing significantly decreased levels of β-catenin compared to the corresponding sFRP4 expression (p < 0.001). Although borderline tumours also showed decreased β-catenin expression compared to sFRP4, this was not significant. In contrast to their sFRP4 expression, adenocarcinomas expressed significantly higher levels of β-catenin, indicating an inverse relationship between β-catenin and sFRP4 expression in benign and malignant mucinous tumours. Furthermore, results also demonstrated a positive trend between sFRP4 expression and tumour grade.

## Discussion

Although the rate of cancer incidence is greater in the breast than the ovaries, the latter has a higher relative mortality. This is because ovarian tumours are often diagnosed at an advanced stage. In addition, a majority of tumours that are initially responsive to chemotherapy eventually acquire drug-resistance to these therapeutic agents [5,6]. Comprehending how this drug resistance occurs is the basis for developing strategies to improve treatment outcomes.

In this study, the five sFRP isoforms were characterized for the first time in a range of chemosensitive and chemoresistant human epithelial ovarian cancer cell lines. Similar to other tumour types, several of the sFRP isoforms were detected in ovarian cancer cells. However, sFRP4 was the dominant isoform in all the cell lines tested. sFRP4 was first isolated from the ovary and it is probable that it may have specificity for this organ [20]; hence, its dominant expression could be indicative of a specific functional role in the ovary. Similarly, sFRP4 mRNA was the only isoform to be differentially expressed between the different cell lines. Western blots and IHC for sFRP4 protein also confirmed that the chemoresistant cells expressed lower levels of this protein. The differential expression of sFRP4 seen in the chemosensitive and chemoresistant cell lines indicated that sFRP4 could serve as a prognostic marker for ovarian tumours.

IHC showed heterogeneous expression of sFRP4 in the various ovarian cell populations examined, in contrast to the pattern of expression in the normal IOSE cells, which was homogenous. The chemosensitive cell line was found to exhibit a larger sub-population of sFRP4 positive cells than the chemoresistant cells. It is known that ovarian tumours have a heterogeneous population of cells. Therefore it is very likely that the cells have a differential response to chemotherapeutic treatment [3,34]. Our results indicated that the heterogeneity of sFRP4 expression was correlated with the response of the cell lines to Cisplatin, with sFRP4 expression as a positive marker for chemosensitivity.

Furthermore, IHC of the cancerous cell lines following treatment with Cisplatin revealed that, compared to untreated controls, both the chemosensitive cell line A2780 and the chemoresistant cell lines lost most of their sFRP4 expressing cells; indicating that Cisplatin selectively targets sFRP4 expressing cells within the heterogeneous population.

Increasing the sFRP4 expression of the chemoresistant cell lines using transfection prior to Cisplatin treatment resulted in more cells expressing sFRP4 and, as predicted, increased their sensitivity to treatment. In contrast, knocking down sFRP4 expression of the chemosensitive A2780 cells by 40% was sufficient to confer these cells with partial resistance to subsequent chemotherapeutic treatment when compared to controls. These results are comparable with a study by He et al., (2005), who reported that significant down regulation of sFRP4 expression promoted cell growth and inhibited chemotherapeutic drug-induced apoptosis in mesothelioma cell lines [35]. Our transfection and silencing experiments confirmed that sFRP4 appeared to have a direct influence on the chemo-response of cancer cells. The data generated from this study present a novel finding and indicate a potential avenue for future research on sFRP4.

The heterogeneous expression of sFRP4 we observed using IHC in our cell lines indicated the presence of at least two sub-populations within ovarian cell cultures. The chemosensitive cells had a larger population of sFRP4 positive cells, and this correlated with their greater sensitivity to treatment. Hypermethylation of the sFRP4 gene has been reported in various cancers and is associated with tumour progression and malignancy [28,29]. We did not examine our ovarian cancer cell lines on whether low sFRP4 expression was associated with hypermethylation of the sFRP4 gene itself, and this would be useful to know.

Our experiments raised some possible explanations for acquired resistance to Cisplatin in human ovarian cancers. From our IHC analysis we know that Cisplatin selectively targets sFRP4 expressing cells, thus continuous treatment would gradually deplete this sub-population of cells over time. Consequently, the resultant population remaining would now be comprised largely of cells that do not express sFRP4 and, hence, be more resistant to subsequent treatment. These data suggest that loss of sFRP4 expression is functionally associated with a more malignant and chemoresistant phenotype, and that treatment itself may select for these cells, thereby resulting in acquired chemoresistance.

Suppression of sFRP4 in the chemosensitive cells resulted in a corresponding increase in β-catenin expression of these cells. This study confirmed the existence of an inverse relationship between sFRP4 and β-catenin expression, and the presence of a signalling interaction between these two proteins in the ovarian cell lines. These findings are consistent with other studies that also reported a similar inverse relationship between these two proteins in endometrium and breast [36,37], and suggests that sFRP4 may act as a tumour suppressor through its interaction with the Wnt/β-catenin signalling pathway by modulating the cellular cytosolic β-catenin pool.

Mucinous tumours, unlike other ovarian epithelial sub-types, have a well characterised progression from benign to borderline and ultimately to adenocarcinomas [38,39]. In addition, mucinous adenocarcinomas, compared to other subtypes, respond poorly to chemotherapy and are known to have a poor prognosis [39]. Similar to our data, a trend has been observed by other studies where down regulation of sFRP4 expression was similarly associated with stage and grade of the cancer [32,40]. These results suggest that the progressive decline of sFRP4 expression in higher grade disease states could be associated with both tumour progression as well as onset of chemoresistance. However, due to our relatively low patient cohort size, this trend needs to be assessed independently in larger patient numbers to further validate the significance of this finding.

## Conclusion

Studies have shown that targeting factors that regulate drug induced apoptosis, cell cycle arrest or inhibit angiogenesis can influence chemosensitivity [41]. Our data are the first to demonstrate that sFRP4 not only plays a key role in the chemo-response of ovarian tumours but can enable these cells to respond better to Cisplatin treatment when up-regulated in chemoresistant cells. Another advantage of sFRP4 is that this molecule can suppress tumourigenic growth either in the presence or absence of the canonical Wnt/β-catenin pathway, as demonstrated in mesothelioma cell lines by Lee et al. (2004) and He et al. (2005) respectively [13,42].

Thus, the results from this study indicate a role for sFRP4 as a predictive marker for ovarian cancer cell chemosensitivity, and suggest that targeting the sFRP4 mediated pathway may be worth pursuing as a novel therapeutic target.

## Methods

### Cell lines

The epithelial ovarian cancer cell lines A2780, A2780-ADR, and A2780-Cis were obtained from the European Collection of Cell Cultures, while the normal epithelial ovarian cell line IOSE was kindly donated to us by Dr. Nelly Auersperg, University of British Columbia, Vancouver, Canada. These cell lines were cultured in RPMI 1640 supplemented with 10% foetal bovine serum, 0.5% L-Glutamine, and 0.5% Penicillin-streptomycin. All cells were cultured at 37°C in a humid incubator with 5% CO_2_.

### RNA extraction and reverse transcriptase PCR

RNA from cells was extracted using Tri-Reagent (Sigma), as per manufacturer’s instructions.

The RNA isolated was then treated with DNase (Promega) before undergoing reverse transcription by heating to 25°C for 10 min, 55°C for 50 min and 70°C for 15 min. After this, cDNA samples were stored at −20°C.

### Quantitative real time PCR

Real time PCR was performed as previously described [16] in a RotarGene 3000 (Corbett Research) using a master mix comprising of SybrIQ (BioRad), sFRP primers and cDNA. The sFRP primer sequences used are listed in Table 1. All PCR data obtained were standardized against expression of GAPDH and β-Actin respectively. Subsequent PCR products were sequenced and the sequence homology of each product was then compared with published sequence using “Blast N” on the Pubmed “BLAST” program.

**Table 1:** Primer sequences, product size and annealing temperature

**Gene**	**Sequence**	**Product size**	**Annealing temp**
sFRP1	*Forward: *ATCTCTGTGCCAGCGAGTTT	202bp	55°C
	*Reverse: *AAGTGGTGGCTGAGGTTGTC		
sFRP2	*Forward: *AGGACAACGACCTTTGCATC	217bp	55°C
	*Reverse: *TTGCTCTTGGTCTCCAGGAT		
sFRP3	*Forward: *AAACTGTAGAGGGGCAAGCA	227bp	55°C
	*Reverse: *GGCAGCCAGAGCTGGTATAG		
sFRP4	*Forward: *CGATCGGTGCAAGTGTAAAA	181bp	54°C
	*Reverse: *GACTTGAGTTCGAGGGATGG		
sFRP5	*Forward: *GATGTGCTCCAGTGACTTTG	352bp	60°C
	*Reverse: *GCAGGGGTAGGAGAACATGA		
GAPDH	*Forward: *CAGAACATCATCCCTGCATCCACT	185bp	59°C
	*Reverse: *GTTGCTGTTGAAGTCACAGGAGAC		
L19	*Forward: *GGACAGAGTCTTGATGATCTC	194bp	51°C
	*Reverse: *CTGAAGGTCAAAGGGAATGTG		
β-actin	*Forward: *GCACCAAGGATGGAGATGTT	173bp	55°C
	*Reverse: *GGACAGAGTCTTGATGATCTC		

### Western blotting

Whole cell lysates and Western blot analysis were performed as previously described with minor modifications [21]. Protein was extracted from cells using radioimmunoprecipitation (RIPA) buffer. The amount of protein was estimated using the Bradford assay (Bradford, 1976). The membranes were probed using anti-mouse β-catenin primary antibody (1:1000) (Cell Signaling, Cat. no. 05–601), Anti-rabbit-sFRP4 (1:750) (Upstate. Cat. No. 09–129), and anti-mouse β-Actin (1:5000) (Sigma. Cat. No. A 5441). Membranes were imaged and quantitated using the Kodak Imagestation 2000MM.

### Immunohistochemistry (IHC)

Cells were grown on 22 mm × 22 mm glass cover slips in 6-well plates in 2 ml of medium containing 100,000 cells per ml. At 24 h fresh medium containing 10 μg Cisplatin or control medium was added and cells were cultured for a further 48 h. The cells were fixed and stained for expression of sFRP4 and β-catenin. The antibodies used were anti-rabbit sFRP4 primary antibody (1:100) and anti-mouse β-catenin primary antibody (1:250). A Nikon (Tokyo, Japan) Eclipse 90i microscope at 40 × objective and a Coolsnap ES camera (Roper Scientific, Duluth, GA) were used for observations and photography of the slides.

### Cell treatment studies

The cells were seeded at density of 50,000 cells per ml (5000 cells per well) onto 96-well plates in 100 μl medium. The cells were allowed to attach for 24 h before 1, 5, and 10 μg/ml concentrations of Cisplatin (Oncotain, Mayne Pharma, VIC, Australia) were added with fresh medium together with a vehicle control (PBS). Cells were further incubated for 24, 48 or 72 h.

### MTS cell viability assay

Cellular viability was assessed by a MTS assay using a Cell Titer 96 Aqueous One solution cell viability kit (Promega, Madison, WI) containing a tetrazolium compound 3-(4,5-dimethylthiazol-2-yl)-5-(3-carboxymethoxyphenyl)-2-(4-sulfophenyl)-2H-tetrazolium (MTS) (yellow), which is reduced by the mitochondria of viable cells to a purple coloured formazan product. Briefly, the cells were seeded and treated. At each time-point 20 μl of MTS reagent was added to the cells and the optical density of the coloured product (i.e. formation of formazan) was measured on a photometric plate reader (Labsystems Multiskan RC) at 490 nm after incubation for 3 h. The absorbance reading for each treatment and time point was calculated and equated against its corresponding control samples in order to determine the effect of Cisplatin on the viability/survival rates of each cell line.

### Quantification of cell death

Quantification of cell death was determined using the JC-1 technique [43] (Invitrogen). At the onset of cell death, the mitochondrial membrane is rapidly depolarized. When the mitochondrial membrane is polarized, the JC-1 dye (5,5^′^,6,6^′^-tetrachloro-1,1^′^,3,3^′^-tetraethyl-benzimidazolyl-carbocyanine iodide) aggregates and fluoresces red. Upon depolarization, JC-1 forms a green fluorescent monomer, so the ratio of aggregated to monomeric JC-1 gives a quantitative representation of the extent of mitochondrial membrane permeability. Positive controls were conducted by the addition of 50 μM FCCP (carbonyl cyanide p-(trifluoromethoxy) phenylhydrazone). Cells undergoing cell death primarily demonstrate green fluorescence while healthy cells fluoresce red/green. JC-1 cell death assays were conducted following Cisplatin treatment for all three time-points (24, 48, 72 h). Plates were analysed using a FluoStar fluorescent plate reader at 520 (green) and 590 nm (red).

### Transient transfections

Cells of chemoresistant cell lines (A2780-ADR and A2780-Cis) were plated in 96-well plates 24 hours before transfection. Lipofectamine 2000 (Invitrogen) was used to mediate transfection using 0.2 μg sFRP4 cDNA construct in pEGFP-N1 plasmid vector (ClonTechInc) (kindly provided by Prof Robert Friis) or empty pEGFP-N1 vector as control as per the manufacturer’s protocol.

### RNA interference

Cells of the chemosensitive cell line A2780 were plated in 96-well plates 24 hours before silencing. RNA interference was conducted using sFRP4 siRNA and non-silencing siRNA control (> 97% pure) purchased from Qiagen-Xeragon (Germantown, MD). The targeted sequence of sFRP4 siRNA is 5-AAGTCCCGCTCATTACAAATT-3, corresponding to +701 to +721 of the human sFRP4 cDNA sequence (the start codon ATG is defined as +1). Following siRNA treatment, the level of sFRP4 in A2780 cells was assessed by performing qPCR and Western blots on mRNA and protein extracted from the cells.

### Tissue microarrays

Archived mucinous ovarian tumour tissue (stored as paraffin embedded tumour blocks) were sourced from the Western Australian Research Tissue Network at St John of God HealthCare SJOGHC), with ethical approval from the SJOGHC Human Research Ethics Committee. Tissue microarrays (TMAs) were prepared as previously described [44]. Immunohistochemical staining for sFRP4 and β-catenin were performed as described previously [45]. The TMAs were probed using anti- β-catenin primary antibody (1:150) (Cell Signaling, Cat. no. 05–601) and anti- sFRP4 (1:100) (Upstate. Cat. No. 09–129). The TMA sections were subjectively classified into one of four categories depending on the percentage and intensity of the cellular staining: negative, weak, moderate, or strong. The sFRP4 and β-catenin expression of all cores (weak, moderate, or strong) for each patient was averaged and quantitated. Next, each tumour core was classified based on their histological subtype (benign, borderline, or adenocarcinoma), and the total sFRP4 and β-catenin expression for each subtype was then quantitated.

### Statistical analysis

Data were analysed using one-way ANOVA and post hoc Dunnett's test with SPSS statistics program version 17.0. Data generated from TMAs were analysed using log rank tests to compare between groups, and chi-squared tests to determine significance between groups. A “p value” of less than 0.05 was considered statistically significant.

## Competing interests

The authors declare that they have no competing interests.

## Authors’ contributions

US performed the quantitative PCR, Western blots, Transfection and silencing experiments, human tissue microarray immunohistochemistry, analysed and interpreted the data, as well as carried out statistical analyses. FA analysed and interpreted data, prepared final figures, and drafted the manuscript. NZ and AD designed the study, analysed and interpreted the data. All authors read and approved the manuscript.

## Supplementary Material

Additional file 1 **Figure S1. **A representative image of a Western blot showing sFRP4 protein expression across the four cell lines. Click here for file

Additional file 2 **Figure S2. **Representative images cut from Western blots demonstrating (A) sFRP4 protein expression was increased in the chemoresistant cell lines following transfection with sFRP4 plasmid; (B) sFRP4 mRNA expression of chemosensitive cell line A2780 was knocked down using siRNA; (C) sFRP4 protein expression of A2780 cells following siRNA treatment. Click here for file

Additional file 3 **Figure S3. **Representative images cut from Western blots demonstrating (A) β-catenin protein expression across the three cell lines; (B) β-catenin protein expression in chemosensitive A2780 cells following siRNA treatment. Click here for file
