# Supplementary material for: Secreted frizzled-related protein 4 expression is positively associated with responsiveness to Cisplatin of ovarian cancer cell lines in vitro and with lower tumour grade in mucinous ovarian cancers
Source: BMC Cell Biol. 2012 Oct 8;13:25. doi: 10.1186/1471-2121-13-25 (PMC3521476; doi:10.1186/1471-2121-13-25)
Supplement: Additional file 1 — Figure S1. A representative image of a Western blot showing sFRP4 protein expression across the four cell lines. [file 1471-2121-13-25-S1.ppt]

## Slide 1
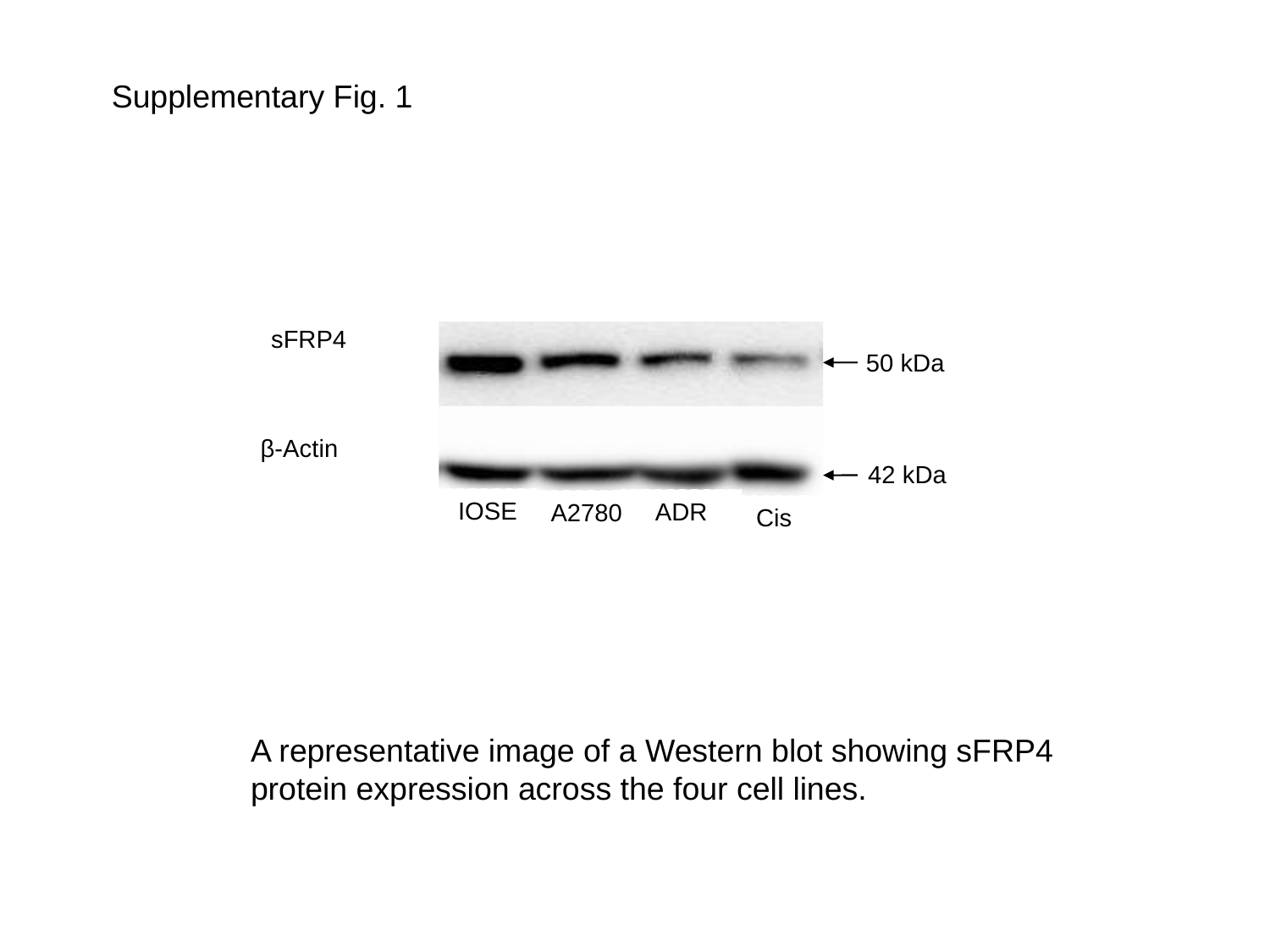

Supplementary Fig. 1
sFRP4
50 kDa
β-Actin
42 kDa
IOSE
ADR
A2780
Cis
A representative image of a Western blot showing sFRP4 protein expression across the four cell lines.
