# Supplementary material for: Secreted frizzled-related protein 4 expression is positively associated with responsiveness to Cisplatin of ovarian cancer cell lines in vitro and with lower tumour grade in mucinous ovarian cancers
Source: BMC Cell Biol. 2012 Oct 8;13:25. doi: 10.1186/1471-2121-13-25 (PMC3521476; doi:10.1186/1471-2121-13-25)
Supplement: Additional file 2 — Figure S2. Representative images cut from Western blots demonstrating (A) sFRP4 protein expression was increased in the chemoresistant cell lines following transfection with sFRP4 plasmid; (B) sFRP4 mRNA expression of chemosensitive cell line A2780 was knocked down using siRNA; (C) sFRP4 protein expression of A2780 cells following siRNA treatment. [file 1471-2121-13-25-S2.ppt]

## Slide 1
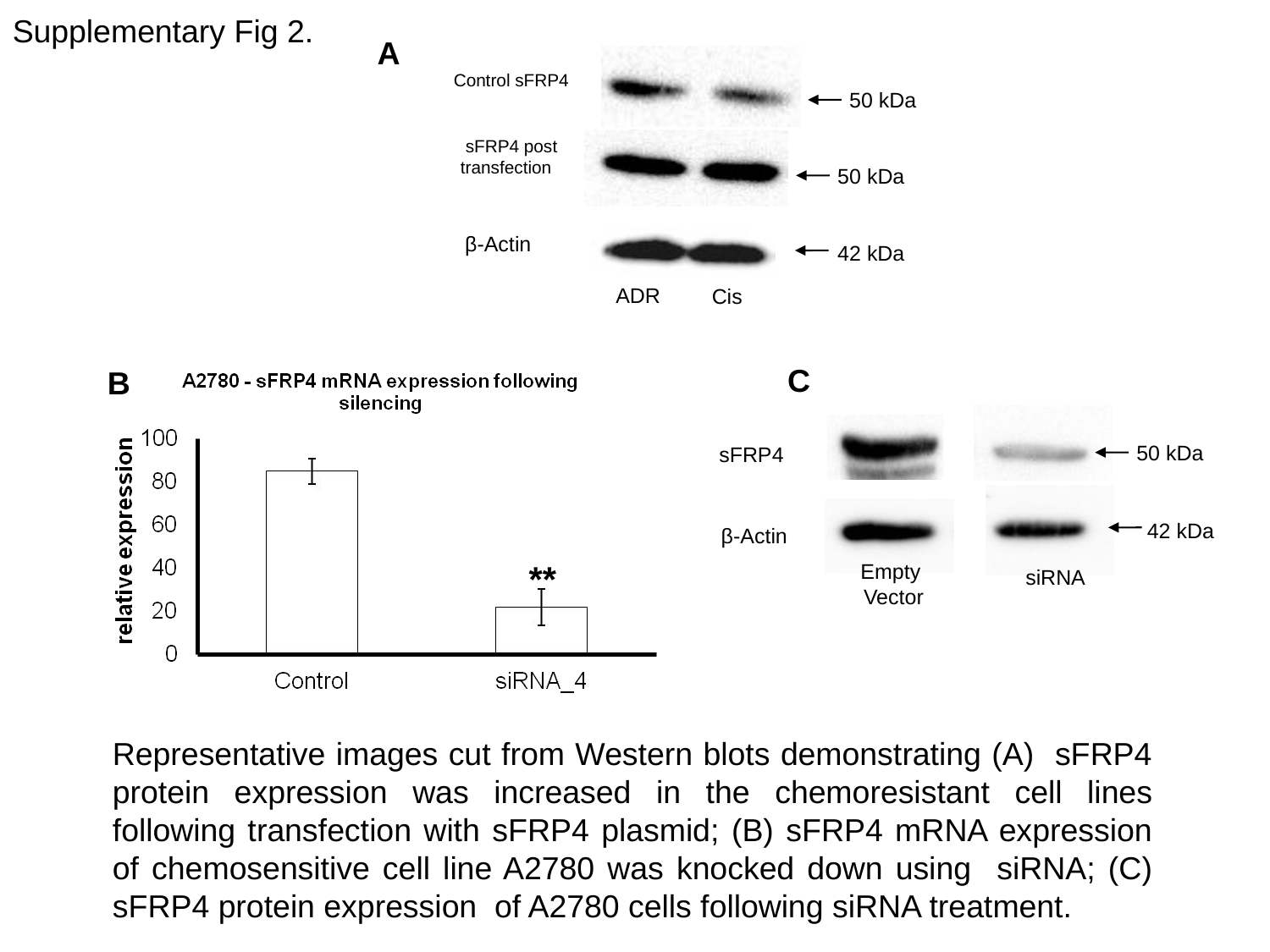

Supplementary Fig 2.
A
Control sFRP4
50 kDa
 sFRP4 post transfection
50 kDa
β-Actin
42 kDa
C
**
50 kDa
sFRP4
42 kDa
β-Actin
Empty
Vector
siRNA
ADR
Cis
B
Representative images cut from Western blots demonstrating (A) sFRP4 protein expression was increased in the chemoresistant cell lines following transfection with sFRP4 plasmid; (B) sFRP4 mRNA expression of chemosensitive cell line A2780 was knocked down using siRNA; (C) sFRP4 protein expression of A2780 cells following siRNA treatment.
