# Supplementary material for: Secreted frizzled-related protein 4 expression is positively associated with responsiveness to Cisplatin of ovarian cancer cell lines in vitro and with lower tumour grade in mucinous ovarian cancers
Source: BMC Cell Biol. 2012 Oct 8;13:25. doi: 10.1186/1471-2121-13-25 (PMC3521476; doi:10.1186/1471-2121-13-25)
Supplement: Additional file 3 — Figure S3. Representative images cut from Western blots demonstrating (A) β-catenin protein expression across the three cell lines; (B) β-catenin protein expression in chemosensitive A2780 cells following siRNA treatment. [file 1471-2121-13-25-S3.ppt]

## Slide 1
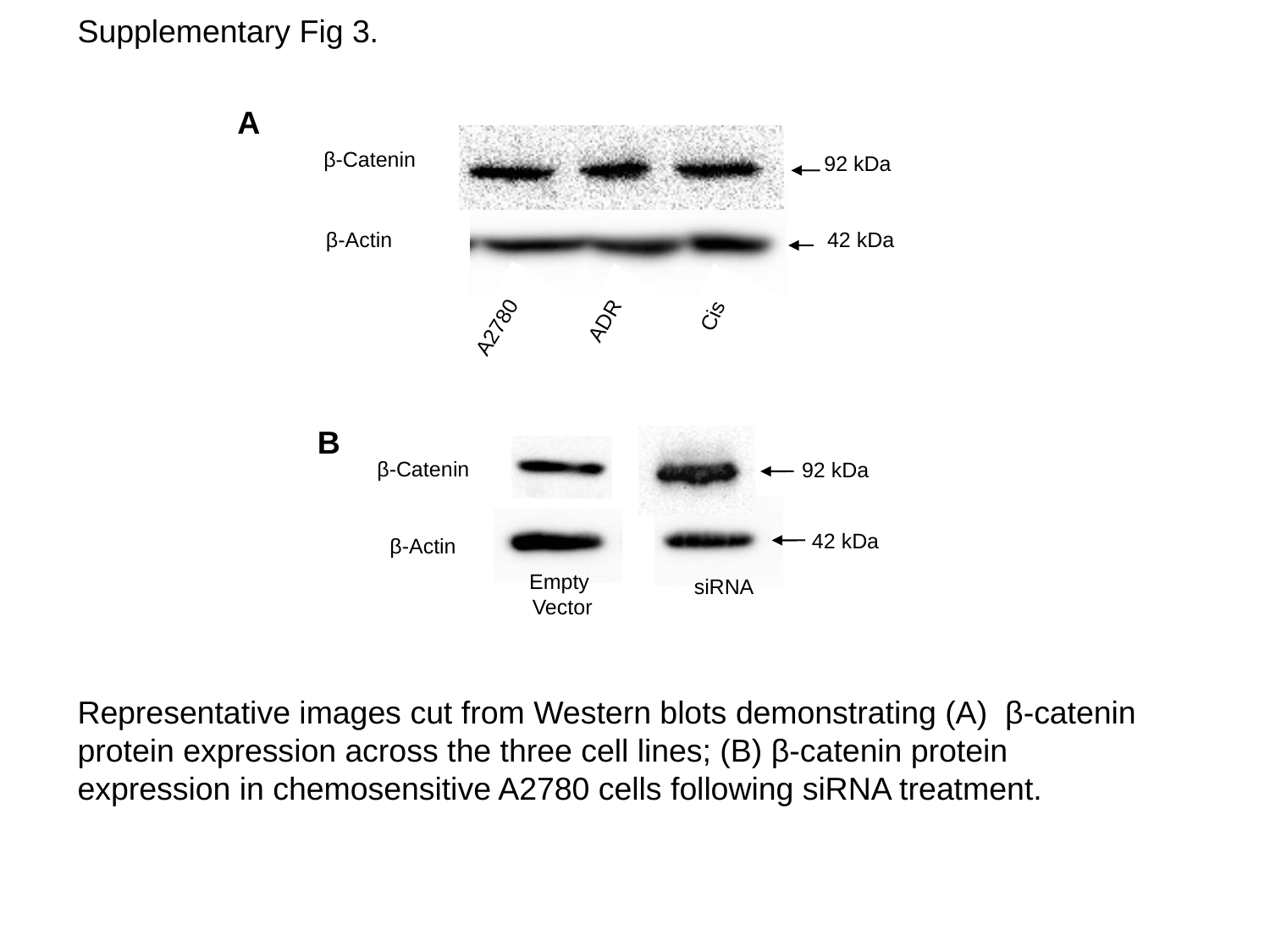

Supplementary Fig 3.
A
β-Catenin
92 kDa
β-Actin
42 kDa
Cis
ADR
A2780
B
β-Catenin
92 kDa
42 kDa
β-Actin
Empty
Vector
siRNA
Representative images cut from Western blots demonstrating (A) β-catenin protein expression across the three cell lines; (B) β-catenin protein expression in chemosensitive A2780 cells following siRNA treatment.
